# Pancreas Procurement and Preservation for Islet Transplantation: Personal Considerations

**DOI:** 10.1155/2011/783168

**Published:** 2011-09-12

**Authors:** Hirofumi Noguchi

**Affiliations:** Department of Gastroenterological Surgery, Okayama University Graduate School of Medicine, Dentistry and Pharmaceutical Sciences, Okayama 700-8558, Japan

## Abstract

Pancreatic islet transplantation is a promising option for the treatment of type 1 diabetic patients. After the successful demonstration of the Edmonton protocol, islet transplantation has advanced significantly on several fronts, including improved pancreas procurement and preservation systems. Since we frequently use pancreata from donors after cardiac death in Japan,we have applied the *in situ* regional organ cooling system for pancreas procurement to reduce the warm ischemic time. To reduce the apoptosis of pancreatic tissue during cold preservation, we have applied the ductal injection of preservation solution. For pancreas preservation, we use modified Kyoto solution, which is advantageous at trypsin inhibition and less collagenase inhibition. In this paper, we show pancreas procurement and preservation in our group for islet transplantation.

## 1. Introduction

Diabetes mellitus is a devastating disease, and over 200 million people are affected worldwide, thus representing about 6% of the world population. Type 1 diabetes results from the autoimmune-mediated destruction of insulin-secreting *β* cells in the islets of Langerhans of the pancreas. Pancreatic islet transplantation represents a viable option for the treatment of patients with unstable type 1 diabetes mellitus with frequent severe hypoglycemia and hypoglycemia unawareness [1, 2]. Recent advances in islet transplantation, including the utilization of donors after cardiac death (DCD) [3–6], single-donor islet transplantation [7–10], and living-donor islet transplantation [11], were based on advanced pancreas transport systems [9, 12, 13], improved islet isolation methods [14–17], enhanced islet engraftment [18–21], and revised immunosuppressant protocols [6, 14, 22]. One of the most important issues affecting islet transplantation is concerned with donor quality [23]. Several critical donor factors have been identified, including donor age, body mass index (BMI), cause of death, usage of vasopressor, hypotensive episode, length of hospitalization, blood glucose levels, transaminases level, creatinine levels, cold preservation time, and procurement team [23–26]. Therefore, effective pancreas procurement and preservation are important for successful islet isolation and transplantation. In this paper, the current advances in pancreas procurement and preservation for islet transplantation in our group are described.

## 2. Pancreas Procurement

Pancreata from donors with brain death (DBD) are procured using a standardized technique to minimize warm ischemia. A preservation solution, such as the University of Wisconsin (UW) solution, is used for *in situ* perfusion of the donor. The human pancreas is excised immediately after the liver and before the kidneys. The first and fourth portions of the duodenum are first divided with a 55 mm linear cutter. The attachment between the retroperitoneal portion and the body of the pancreas is then dissected toward the spleen. The superior surface of the pancreas is divided toward the spleen, and the short gastric arteries and vein are dissected until the stomach is separated from the spleen. The pancreas is then rapidly excised *en bloc* together with the spleen. The spleen and duodenum are subsequently removed on a back table, and a cannula is inserted into the main pancreatic duct. 

**Figure 1:**
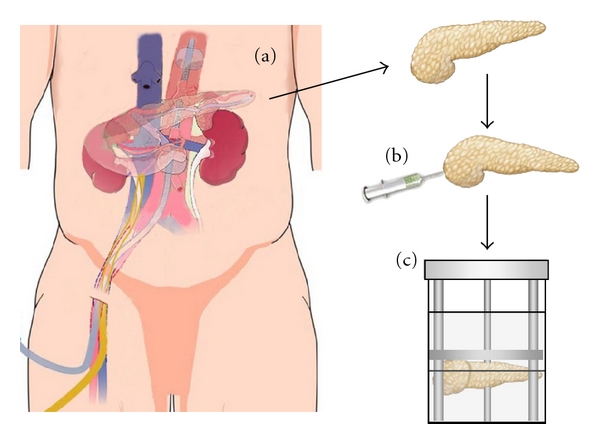
Pancreas procurement and preservation for islet transplantation. (a) *In situ* regional cooling system to DCD pancreata. (b) Ductal injection of preservation solution. (c) Pancreas preservation by MK solution/PFC two-layer method.

Currently, only a few clinical studies have reported that islet transplantation from DCD is possible to treat type 1 diabetes [27, 28]. Vasopressors are used for most DCDs, and they tend to have hypotensive episodes, long term hospitalization, and high levels of blood glucose, transaminases, and creatinine which have been identified as critical factors that affect the quality of the pancreas [23–26]. Islet transplantation from DCD is particularly important for countries such as Japan, where the isolation of islets from pancreata of donors who are classified as brain-dead but whose hearts are beating is prohibited by law. We developed the novel procurement technique in collaboration with the kidney procurement team [29]. After confirmation of brain death, a double-balloon catheter is inserted to prevent ischemic damage to the human pancreas by using an *in situ* regional organ cooling (ISRC) system that was originally developed for procurement of the kidney [30]. Before cardiac arrest, a tip of the double-balloon catheter is placed above the celiac axis in the aorta via the femoral artery and only a few centimeters above the location used for an ordinary nephrectomy for procurement from a DCD. A venous catheter is also placed in the inferior vena cava via the femoral vein for drainage of the perfusate and blood. ISRC for the pancreas and kidney (ISRC-PK) is achieved by pump or drip infusion (drip speed 20 mL/min) of a hypothermic lactated ringer solution beginning immediately after cardiac arrest and then continuing until the end of the nephrectomy and pancreatectomy. After laparotomy, the lesser sac is opened by dividing the gastrocolic and gastrohepatic ligaments to determine whether the pancreas has uniform perfusion efficacy by means of ISRC-PK. Perfusion of the pancreas is evaluated by the uniform color change of the pancreas and the coldness of the pancreas surface after laparotomy. After a visual check of the pancreas, 500 mL of sterile crushed ice is placed on it to avoid warm ischemic injury and then the pancreas is harvested (Figure 1(a)) [29]. 

The ISRC system was originally developed for the procurement of the kidney [30], and the only modification we made is the position of the double-balloon catheter to ensure both pancreas and kidney protection. This ISRC system reduced the warm ischemic time to only 3 minutes on average [29]. We have used lactate ringer solution instead of UW solution for perfusion. Lactate ringer solution has a low potassium concentration and low viscosity in comparison to UW solution. A low potassium concentration could prevent potassium-induced vasospasms while a low viscosity helps to induce rapid perfusion. Therefore, using lactate ringer for perfusion might be important in ISRC.

## 3. Ductal Injection of Preservation Solution

We previously developed a new method for large-scale porcine islet isolation from market-weight pigs [31], based on a report by O'Neil et al. [32]. Although some steps of the new method seemed technically inferior to the standard automated method using a Ricordi chamber, islet yield per gram for our new method was relatively higher (but not significantly so) than that for the Ricordi method [33]. It is possible that the advantage of the new method was the injection of 1.0–1.5 mL/g pancreas of UW-D (UW solution with high Na^+^/low K^+^) solution. It was also shown that islet yields from pancreata with intraductal flush, along with collagenase prior to preservation, were superior to vascular flush [34]. We speculate that the ductal injection of a large volume of preservation solution (1 mL/g pancreas) may improve the islet yield. We investigated whether ductal injection (UW and modified Kyoto (MK) solution) before pancreas storage improves the islet yields in islet isolation using porcine pancreata. After obtaining the pancreas, we immediately inserted a cannula into the main pancreatic duct, infused a large amount of preservation solution (1 mL/g pancreas) for ductal protection, and placed the pancreas into a preservation container (Figure 1(b)). The islet yield both before and after purification was significantly higher in the ductal injection group than in the control group. The TUNEL-positive cells in the ductal injection group significantly decreased in comparison to the control group. The ductal injection of preservation solution increased the ATP level in the pancreas tissue and reduced trypsin activity during the digestion step. In a transplant model, ductal injection improved the islet graft function. These findings suggest that the ductal injection of preservation solution leads to improved outcomes for pancreatic islet transplantation [35]. Another group also showed the ductal injection of a small volume (0.05–0.1 mL/g) of UW solution at the time of pancreas procurement to improve the islet yield and function in a rodent model [36]. Based on these data, we now use this technique for clinical islet transplantation. With this technique, we rarely see clumping or DNA release, even using DCD, and we never use DNase.

## 4. Preservation Solution

UW solution has been recognized as the gold standard solution for organ preservation. UW solution is used extensively as a cold storage solution during procurement and transport of the pancreas prior to islet isolation. However, UW solution has several disadvantages: it must be stored in the cold until use, and its short shelf life makes it expensive. It is also highly viscous, which may complicate the initial organ flush [37]. For islet isolation, it has been observed that UW inhibits the collagenase digestion phase of islet isolation, thus resulting in poor islet yields and islets of poor viability [38, 39]. It has been reported that the components in UW solution found to be most inhibitory were magnesium, low Na^+^/high K^+^, hydroxyethyl starch (HES), and adenosine. Furthermore, previous reports also indicated that allopurinol in combination with either lactobionate or glutathione markedly inhibited collagenase and that the most inhibitory solution tested was a combination of three components, raffinose, glutathione, and lactobionate [39]. We evaluated the effect of MK solution for islet isolation [12]. Kyoto University developed the ET-Kyoto solution, and its effectiveness in cold lung storage has been demonstrated in clinical lung transplantation [40, 41]. It also is effective for skin flap storage, and its clinical application is beginning in this field [42]. MK solution is a modified ET-Kyoto solution, in which ulinastatin is added. MK solution contains trehalose, gluconate, and ulinastatin as distinct components. Trehalose has a cytoprotective effect against stress, and gluconate acts as an extracellular oncotic agent, which prevents cells from swelling [44]. Ulinastatin is a trypsin inhibitor and eliminates trypsin activity during pancreas preservation [12]. Due to the chemical stability of the effective components and other ingredients, MK solution, but not UW solution, can be stored at room temperature for a long period. MK solution has high Na^+^/low K^+^, and it includes only HES at a lower concentration than UW solution, thus suggesting a lower collagenase inhibition than UW solution. It has also been shown that the Na^+^/K^+^ ratio, adenosine, allopurinol, and glutathione are not essential for the cold storage of pancreatic digest prior to islet purification [45]. Moreover, trehalose and ulinastatin inhibit collagenase digestion less than UW solution [12]. The high potassium concentration in UW solution causes vasospasms and insulin release from pancreatic *β* cells [46], and the high viscosity of UW solution may thus prevent sufficient flushing and ductal injection. In both porcine and human islet isolation, the islet yield was significantly higher in the MK group compared with the UW group [12, 47]. These findings show that MK solution is a more effective cold-storage solution in pancreas preservation for islet isolation than UW solution.

We next compared histidine-tryptophan-ketoglutarate (HTK) solution and MK solution for islet isolation. HTK solution was originally developed for cardioplegia and is being used with increasing frequency in cardiac, renal, and hepatic transplantation [48, 49]. The protective effect of HTK solution is based on the strong buffering capacity of histidine. This solution has a low viscosity, easy handling properties, and a relatively low cost. Some studies have demonstrated similar results between UW and HTK solutions for pancreas preservation, in not only experimental animal models [43, 50, 51] but also clinical pancreas transplantation [52–54]. We used HTK solution with ulinastatin (modified HTK solution (M-HTK)) in this study because MK solution includes ulinastatin. In porcine islet isolation, the islet yield after purification was significantly greater in the MK group than in the M-HTK group. The MK group had a significantly higher ATP level in the islets than in the M-HTK group. These data suggest that MK solution is better for pancreas preservation before islet isolation than M-HTK solution [55]. The M-HTK solution includes magnesium but does not include HES, adenosine, allopurinol, lactobionate, glutathione, or raffinose. There are no significant differences between the MK and M-HTK solutions regarding collagenase activity. Therefore, the different islet yields after purification are not due to differences in collagenase inhibition between these two solutions. Since a significantly higher ATP level in islets was observed in the MK group compared to the M-HTK group, the cytoprotective effect such as HES and/or trehalose might be a factor in the islet yield differences observed between the two solutions. Another group recently reported that, compared with UW solution, HTK solution has similar efficiency for preserving human pancreata for subsequent islet isolation during <10 h cold ischemia time, but prolonged cold storage resulted in a reduced islet yield [56]. 

Recently, Celsior solution has been used as an alternative solution for organ preservation. Celsior is an extracellular solution deprived of colloid and was initially developed for heart preservation [57]. Preliminary clinical studies showed no differences between UW and Celsior for lung [58], liver [59], and kidney [60] preservation. Hubert et al. recently reported on the application of Celsior solution for *in situ* perfusion of the donor before human and pig islet isolation [61]. Their data showed the *in situ* perfusion of UW solution to be superior to Celsior solution. In contrast to UW, Celsior induced cell swelling and pancreas edema after only four hours of cold storage. The components of Celsior solution are in part similar to MK solution (a high-sodium/low-potassium composition with comparatively low viscosity) and in part similar to UW solution (including lactobionate acid and glutathione). We next compared modified Celsior solution (Celsior solution with HES and nafamostat mesilate, HNC) and MK solution [47]. Since Celsior solution lacks HES, which is an oncotic agent and protects cell swelling, we added HES to Celsior solution in this study. We also added nafamostat mesilate, one of the trypsin inhibitors, to Celsior because one of the advantages of MK solution is trypsin inhibition by ulinastatin. Nafamostat mesilate has a higher level of trypsin inhibition than ulinastatin [62, 63]. In human islet isolation, the islet yield after purification was significantly higher in the MK group than in the HNC group. The HNC group had a longer phase I period (digestion time), a higher volume of undigested tissue, and a higher percentage of embedded islets, thus suggesting that the solution may inhibit collagenase. However, there was no significant difference in the ATP content in the pancreata or in the attainability of posttransplant normoglycemia in diabetic nude mice between the two groups, thus suggesting that the quality of islets was similar between the two groups. These data suggest that MK solution is better for pancreas preservation before islet isolation than HNC solution (Tables 1 and 2).

## 5. Trypsin Inhibitors in Preservation Solution

Trypsin from pancreatic acinar cells destroys islets. Previous study has shown that trypsin inhibition by Pefabloc during human pancreas digestion improves islet yield and reduces the fraction of embedded (trapped) islets [64], thus suggesting that trypsin may degrade the ductules and thus reduce the delivery of collagenase solution to tissue around the islets. We previously reported that pancreas preservation using MK solution including ulinastatin, which eliminated trypsin activity during pancreas preservation, was superior to that using ET-Kyoto solution without the trypsin inhibitor in a rat model [12]. Furthermore, the advantages of MK solution are its trypsin inhibition and less collagenase inhibition in human and porcine islet isolation [12, 47]. Therefore, we compared ulinastatin with other trypsin inhibitors, including Pefabloc, gabexate mesilate, and nafamostat mesilate, in preservation solution for porcine islet isolation [62, 63]. Trypsin inhibition is greater in ET-Kyoto with gabexate mesilate (GK) solution and ET-Kyoto with nafamostat mesilate (NK) solution than in MK solution. The islet yield before purification was higher in the MK group than in the ET-Kyoto with Pefabloc (PK) group. Viability was higher for the MK group than for either the GK group or the NK group. The stimulation index was higher for the MK group than for either the PK group or the GK group. These data suggest that MK solution was synthetically superior to the PK, GK, or NK solutions, although trypsin inhibition is greater in GK and NK solutions than in MK solution (Table 3) [62, 63], possibly due to differences of inhibitory effects of cytokines. Ulinastatin has been shown to inhibit not only trypsin activity but also the release of neutrophil elastase. It also downregulates transcription of TNF mRNA, the activation of endothelial cells, and the expression of ICAM-1 induced by endotoxin *in vitro* [65–67]. The administration of ulinastatin has been shown to decrease ischemia-reperfusion injury [68] or attenuate the elevation in the concentrations of inflammatory cytokines and C-reactive protein, a marker of inflammation [69], in the transplanted small intestine.

Recently, the importance of tryptic-like activity (TLA) obtained from *Clostridium histolyticum* in collagenase NB1 with neutral protease for efficient islet isolation was demonstrated [70]. Enhancing TLA resulted in a significant reduction of recirculation time and incrementally increased human islet yield. The clostridial TLA and pancreatic trypsin seemed to be different in their specificity toward islet and nonislet pancreatic tissue because no detrimental effect on islet viability and integrity was detected on clostridial TLA. If trypsin inhibitors inhibit clostridial TLA as well as pancreatic trypsin, then they may inhibit pancreas digestion. This may explain the synthetic superiority of MK solution to PK, GK, or NK solutions, although trypsin inhibition is greater in GK and NK solutions than in MK solution (Figure 1(c)).

## 6. Two-Layer Method

The two-layer preservation method (TLM), which uses the concept of normobaric oxygenation comprising a cold organ preservation solution (UW solution) with a perfluorochemical (PFC) oxygen carrier solution, with the pancreas being suspended between the two immiscible layers, has been utilized for many clinical trials of islet transplantation [71–74]. However, two recent large-scale studies showed no beneficial effect of TLM, compared with UW storage, on human islet isolation and transplantation [75, 76]. We reevaluated the effect of TLM using three different groups: group 1: UW simple storage; group 2: TLM performed by multiorgan procurement teams (not specialists in islet isolation); group 3: TLM performed by specialists in islet isolation. There were no significant differences between group 1 and 2, whereas islet yields were significantly higher for group 3 compared with either groups 1 or 2. Our data suggest that performance of TLM by experts could improve the outcome of islet isolation and transplantation [71].

On the other hand, Papas et al. showed that the oxygen penetration depth is about 1 mm, suggesting that pancreas oxygenation is limited during preservation with the TLM [77]. In other words, their data suggest that the percentage of pancreas oxygenation by TLM depends on its thickness and the trimming of the pancreas before preservation by TLM is thus considered to be important for pancreas oxygenation.

## 7. Conclusion

ET-Kyoto with ulinastatin was the best combination for pancreas preservation in our studies. Since one of the advantages of MK solution is less collagenase inhibition in islet isolation, it is also suitable for ductal injection. Based on these data, we now use the *in situ* regional cooling system for DCD pancreata, the ductal injection of preservation solution, and pancreas preservation by MK solution during clinical islet isolation/transplantation. The* in situ* regional cooling system to DCD pancreata, ductal injection, and preservation by MK solution is therefore considered to be useful improvement that may help to increase organ utilization and thereby achieve good outcomes after islet transplantation.

##  Conflict of Interest

The author of this manuscript has no conflict of interests.

## Figures and Tables

**Table 1:** Composition and other characteristics of each preserving solution.

	ET-K	MK	UW	HNC	M-HTK
Na (mmol/L)	100	100	29	100	15
K (mmol/L)	43.5	43.5	125	15	10
Mg (mmol/L)	—	—	5	13	4
Ca (mmol/L)	—	—	—	0.25	0.015
Cl (mmol/L)	—	—	—	41	50
Gluconate (mmol/L)	100	100	—	—	—
Phosphate (mmol/L)	25	25	25	—	—
Sulfate (mmol/L)	—	—	5	—	—
Lactobionate (mmol/L)	—	—	100	80	—
Raffinose (mmol/L)	—	—	30	—	—
Trehalose (mmol/L)	120	120	—	—	—
Adenosine (mmol/L)	—	—	5	—	—
Alloprinol (mmol/L)	—	—	1	—	—
Glutathione (mmol/L)	—	—	3	3	—
HES (g/L)	30	30	50	30	—
Ulinastatin (×10^3^ U/L)	—	100	—	—	100
Nafamostat mesilate (mg/L)	—	—	—	20	—
Histidine (mmol/L)	—	—	—	30	198
Mannitol (mmol/L)	—	—	—	60	30
*α*-ketoglutarate (mmol/L)	—	—	—	—	1
Tryptophan (mmol/L)	—	—	—	—	2
Glutamic acid (mmol/L)	—	—	—	20	—
Osmolality (mOsm)	366	366	320	355	310

HES: hydroxyethyl starch; ROS: reactive oxygen species; ET-K: ET-Kyoto solution; MK: modified ET-Kyoto solution; UW: University of Wisconsin solution; HNC: Celsior solution with HES and nafamostat mesilate; M-HTK: modified histidine-tryptophan-ketoglutarate solution.

**Table 2:** Comparison between the different preservation solutions.

Comparison	Superior	Human/animal study	Reference
MK versus UW	MK	Porcine	[12]
MK versus UW	MK	Human	[41] (in discussion)
MK versus ET-K	MK	Rat	[12]
MK versus M-HTK	MK	Porcine	[43]
MK versus HNC	MK	Human	[41]

UW: University of Wisconsin solution;MK: modified ET-Kyoto solution (ET-Kyoto solution with ulinastatin); ET-K: ET-Kyoto solution; M-HTK: modified histidine-tryptophan-ketoglutarate solution (HTK solution with ulinastatin); HNC: Celsior solution with HES and nafamostat mesilate.

**Table 3:** Trypsin inhibitors in ET-Kyoto solution.

	Trypsin	Isle yield	Viability	SI*
Trypsin inhibitors	inhibition	versus ulinastatin	versus ulinastatin	versus ulinastatin
Ulinastatin	++	—	—	—
Pefabloc	+	Lower	n.s.	Lower
Gabexate mesilate	+++	n.s.	Lower	Lower
Nafamostat mesilate	++++	n.s.	Lower	n.s.

*Stimulation index; n.s.: not significant.
